# Acquired resistance to temsirolimus is associated with integrin α7 driven chemotactic activity of renal cell carcinoma *in vitro*

**DOI:** 10.18632/oncotarget.24650

**Published:** 2018-04-10

**Authors:** Tobias Engl, Jochen Rutz, Sebastian Maxeiner, Sorel Fanguen, Eva Juengel, Sebastian Koschade, Frederik Roos, Wael Khoder, Igor Tsaur, Roman A. Blaheta

**Affiliations:** ^1^ Department of Urology, Goethe-University, Frankfurt am Main, Germany; ^2^ Current address: Department of Urology and Pediatric Urology, University Medical Center Mainz, Mainz, Germany; ^3^ Department of Medicine II, Hematology and Oncology, Goethe-University, Frankfurt am Main, Germany

**Keywords:** renal cell cancer, temsirolimus, resistance, chemotaxis, ITGA7

## Abstract

The mechanistic target of the rapamycin (mTOR) inhibitor, temsirolimus, has significantly improved the outcome of patients with renal cell carcinoma (RCC). However, development of temsirolimus-resistance limits its effect and metastatic progression subsequently recurs. Since integrin α7 (ITGA7) is speculated to promote metastasis, this investigation was designed to investigate whether temsirolimus-resistance is associated with altered ITGA7 expression in RCC cell lines and modified tumor cell adhesion and invasion. Caki-1, KTCTL-26, and A498 RCC cell lines were driven to temsirolimus-resistance by exposing them to temsirolimus over a period of 12 months. Subsequently, adhesion to human umbilical vein endothelial cells, to immobilized fibronectin, or collagen was investigated. Chemotaxis was evaluated with a modified Boyden chamber assay and ITGA7 expression by flow cytometry and western blotting. Chemotaxis significantly decreased in temsirolimus-sensitive cell lines upon exposure to low-dosed temsirolimus, but increased in temsirolimus-resistant tumor cells upon reexposure to the same temsirolimus dose. The increase in chemotaxis was accompanied by elevated ITGA7 at the cell surface membrane with simultaneous reduction of intracellular ITGA7. ITGA7 knock-down significantly diminished motility of temsirolimous-sensitive cells but elevated chemotactic activity of temsirolimus-resistant Caki-1 and KTCTL-26 cells. Therefore, ITGA7 appears closely linked to adhesion and migration regulation in RCC cells. It is postulated that temsirolimus-resistance is associated with translocation of ITGA7 from inside the cell to the outer surface. This switch forces RCC migration forward. Whether ITGA7 can serve as an important target in combatting RCC requires further investigation.

## INTRODUCTION

In the past years therapy for metastatic renal cell carcinoma (RCC) has changed. Better understanding of the molecular biology of RCC has led to the development of several targeted therapeutic agents. The Phosphatidyl-Inositol-3 Kinase (PI3K)-Akt-mTOR pathway has been identified as a pivotal key regulator of cell growth, cell proliferation, and cell survival. Activation of PI3K-Akt-mTOR is a characteristic feature of RCC and other solid tumors and results in disturbed cell cycle control, ultimately leading to cell de-differentiation, proliferation and metastatic dissemination. Hence, targeting the mTOR pathway has been proposed as an innovative strategy to fight cancer.

The development of drugs targeting mTOR has led to significant improvement in RCC prognosis. The mTOR inhibitor, temsirolimus (Torisel®), has been approved for first-line treatment of RCC patients with poor-prognosis, whereas the oral mTOR-inhibitor RAD001, (everolimus; Afinitor®), is recommended for patients with advanced progressive RCC or for patients with failed VEGF-targeted therapy. Clinical studies have demonstrated a significant benefit of both mTOR-inhibitors in treating RCC patients (summarized in [1]).

Unfortunately, not all patients benefit equally well from an mTOR based regimen, and disease progression inevitably occurs during treatment. Based on clinical data, it has been argued that chronic drug exposure triggers the development of resistance, ultimately limiting mTOR-inhibitor utility. Particularly, the mTOR related proteins Akt and S6K1 have been shown to be re-activated under long-term everolimus exposure [2]. In addition, histone modifications and elevation of cdk/cyclin members may also speed up the mitotic cycle [3, 4].

Evidence that integrin adhesion receptors are involved in resistance development has been presented. Integrins are transmembrane cell surface molecules composed of specific α and β subunits. They are intricately involved in cell-cell and cell-matrix contact and are important regulators of cell adhesion and migration. During neoplastic dedifferentiation, integrins modify intracellular signaling pathways, enabling cells to begin metastatic progression.

Recently, evidence has been provided that temsirolimus non-responsiveness is associated with altered integrin α5 and β3 expression levels, coupled to a functional change in the integrin molecules, driving tumor cells towards high motility [5]. Novel data point to the integrin subtype α7 (ITGA7) as a potential tumor biomarker [6, 7] and it has been speculated that ITGA7 could serve as a highly specific diagnostic and therapeutic target [8, 9]. Still, it is not yet clear whether temsirolimus induced drug resistance contributes to changes in ITGA7 expression or whether alterations of this receptor type can modify invasive properties of RCC cells. Therefore, an *in vitro* investigation utilizing three RCC cell lines with and without acquired resistance towards temsirolimus is presented here to compare ITGA7 expression and ITGA7 driven RCC adhesion and migration.

## RESULTS

### Resistance to temsirolimus causes elevated tumor cell adhesion to HUVEC

**Figure 1:**
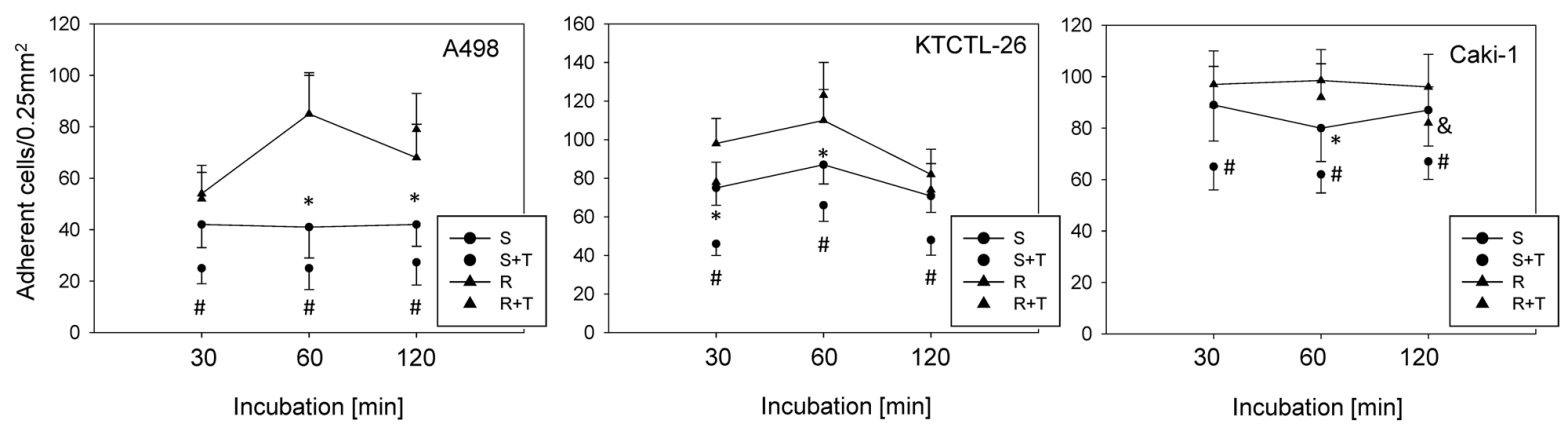
Adhesion of A498, KTCTL-26, and Caki-1 cells to HUVEC From each cell line four cell cultures were primed by receiving fresh medium for 3 days, which were then introduced to a HUVEC monolayer: sensitive (S) cells, sensitive cells+temsirolimus (S+T) by exposing to 10 nM/ml temsirolimus, resistant (R) cells, resistant cells+temsirolimus (R+T) by reexposure to 10 nM/ml temsirolimus. Resistance to temsirolimous had been induced over a period of 12 months. One of six separate experiments is shown. ^*^ indicates significant difference between sensitive (S) and resistant (R) cells, # indicates significant difference between sensitive (S) and sensitive+temsirolimus (S+T), & indicates significant difference between resistant (R) and resistant+temsirolimus (R+T).

Temsirolimus-resistant cells displayed increased adhesion of Caki-1, KTCTL-26, and A498 cells to a HUVEC monolayer (Figure 1) compared to temsirolimus-sensitive cells over a period of 2 h. Exposing the sensitive cell lines to 10 nM/ml temsirolimus induced a significant down-regulation of adhesion, whereas reexposure of the resistant cell lines to 10 nM/ml temsirolimus did not significantly alter cell adhesion in two of the cell lines: KTCTL-26 and A498. A significant temsirolimus induced down-regulation in the temsirolimus-resistant Caki-1 cells did become apparent after 120 min incubation.

### Tumor cell binding to the extracellular matrix proteins, collagen and fibronectin

**Figure 2:**
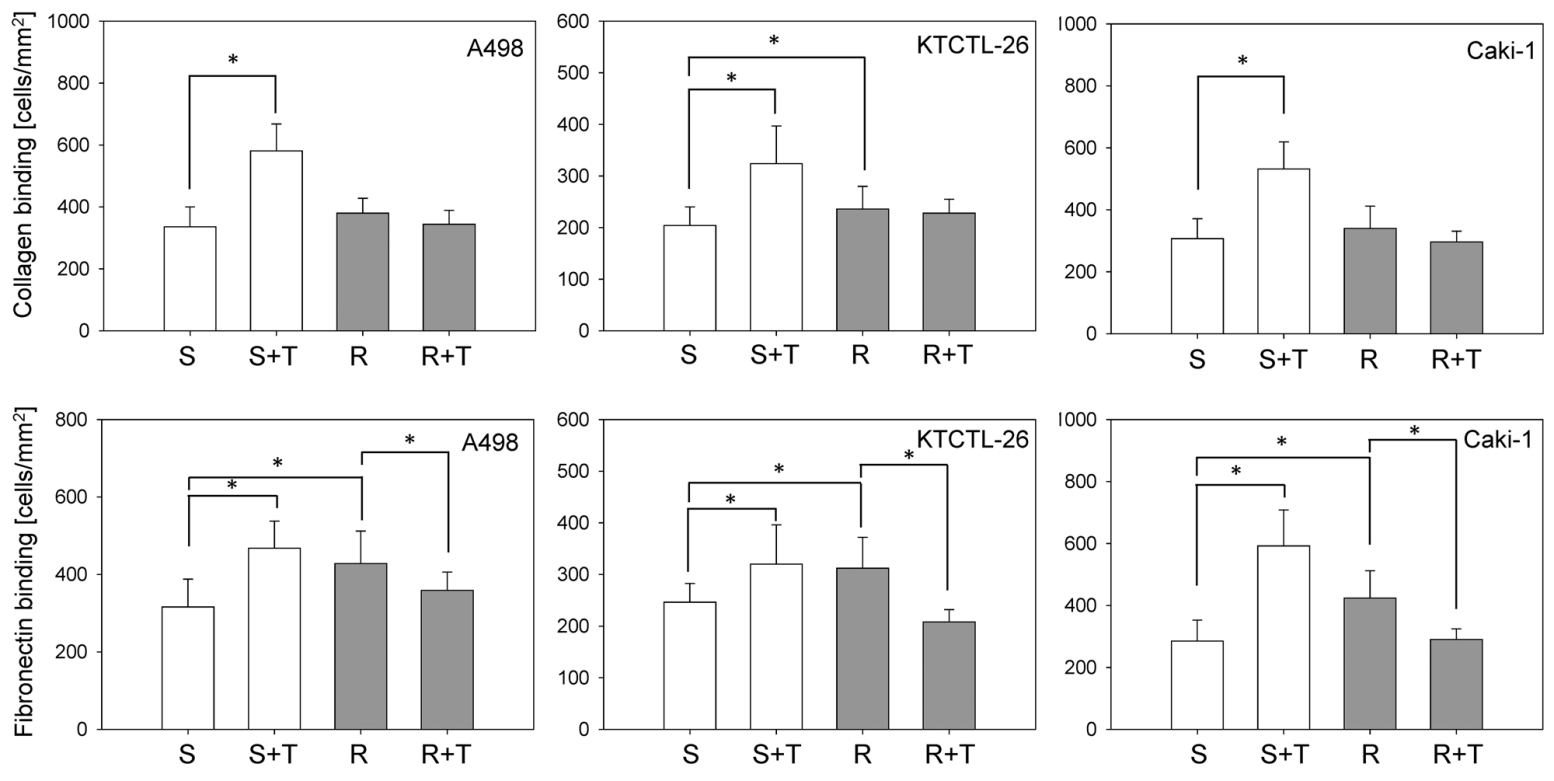
Adhesion of A498, KTCTL-26, and Caki-1 cells to collagen and fibronectin From each cell line four cell cultures were primed by receiving fresh medium for 3 days, which were then introduced to immobilized collagen or fibronectin for 60 min: sensitive (S) cells, sensitive cells+temsirolimus (S+T) by exposing to 10 nM/ml temsirolimus, resistant (R) cells, resistant cells+temsirolimus (R+T) by reexposure to 10 nM/ml temsirolimus. Resistance to temsirolimous had been induced over a period of 12 months. Mean values were calculated from five counts. One representative of six experiments is shown. ^*^indicates significant differences.

Collagen binding was not distinctly modified in the resistant versus sensitive cell lines. However, exposing sensitive Caki-1, KTCTL-26, and A498 cells to temsirolimus significantly enhanced the number of attached cells (Figure 2 upper graphs). Reexposing resistant cell lines to 10 nM/ml temsirolimus did not significantly alter cell binding, compared to resistant cells not reexposed to temsirolimus.

In all three cell lines significantly more temsirolimus-resistant tumor cells attached to fibronectin, compared to the sensitive cell lines. Reexposing resistant cells to 10 nM/ml temsirolimus led to a significantly down-regulated tumor cell binding, compared to resistant cells not reexposed to temsirolimus. This was in contrast to the behavior of sensitive cells, which was significantly up-regulated when they were exposed to temsirolimus, compared to sensitive cells not exposed to temsirolimus (Figure 2 lower graphs).

### Resistance alters chemotactic tumor cell behavior

**Figure 3:**
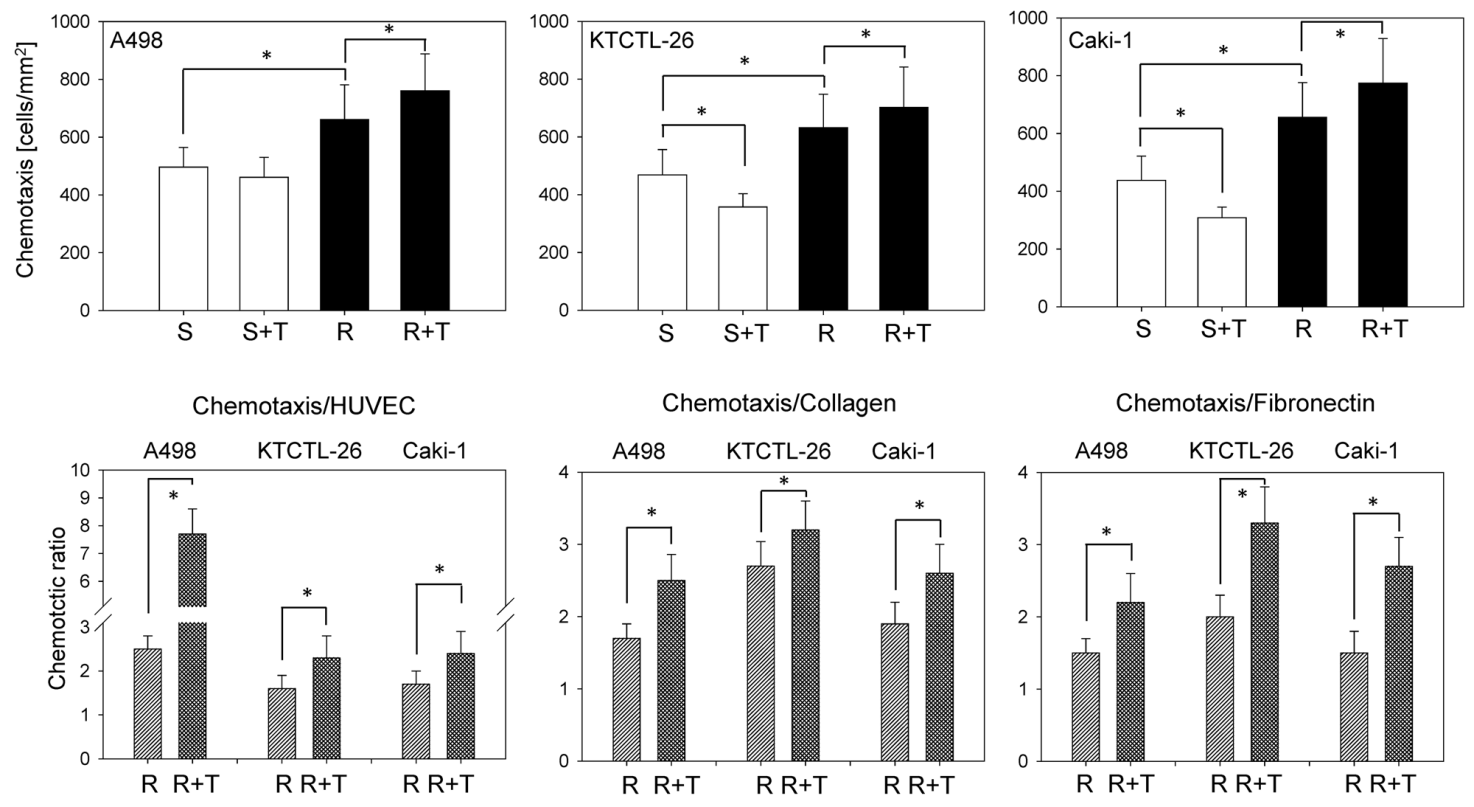
Temsirolimus resistance alters RCC chemotaxis From each cell line four cell cultures were primed by receiving fresh medium for 3 days and subsequently subjected to the Transwell chamber assay: sensitive (S) cells, sensitive cells+temsirolimus (S+T) by exposing to 10 nM/ml temsirolimus, resistant (R) cells, resistant cells+temsirolimus (R+T) by reexposure to 10 nM/ml temsirolimus. Cells moving to the lower surface of the membrane were stained using hematoxylin and counted. Mean values were calculated from five counts. One representative of six experiments is shown. The quotient between the number of cells which adhered to either HUVEC, collagen, or fibronectin and the number of migrated cells is shown in the lower panel and compares resistant tumor cells (R) to resistant tumor cells re-exposed to 10 nM temsirolimus (R+T). ^*^indicates significant difference.

Chemotaxis became elevated under chronic temsirolimus exposure (resistant), compared to the sensitive cells. Application of temsirolimus to the sensitive cells significantly reduced the number of chemotactic Caki-1 and KTCTL-26 (but not A498 cells; Figure 3 upper), whereas the number of resistant chemotactic cells was increased following reexposure to 10 nM/ml temsirolimus. Further analysis revealed a significant enhancement of the quotient between the number of chemotactic cells and the number of cells attached to HUVEC or bound to collagen or fibronectin (resistant cells exposed to 10 nM/ml temsirolimus versus resistant cells not re-exposed to 10 nM/ml temsirolimus) (Figure 3 lower). An elevated quotient implies more bound cells capable of chemotactic movement.

### ITGA7 expression

**Figure 4:**
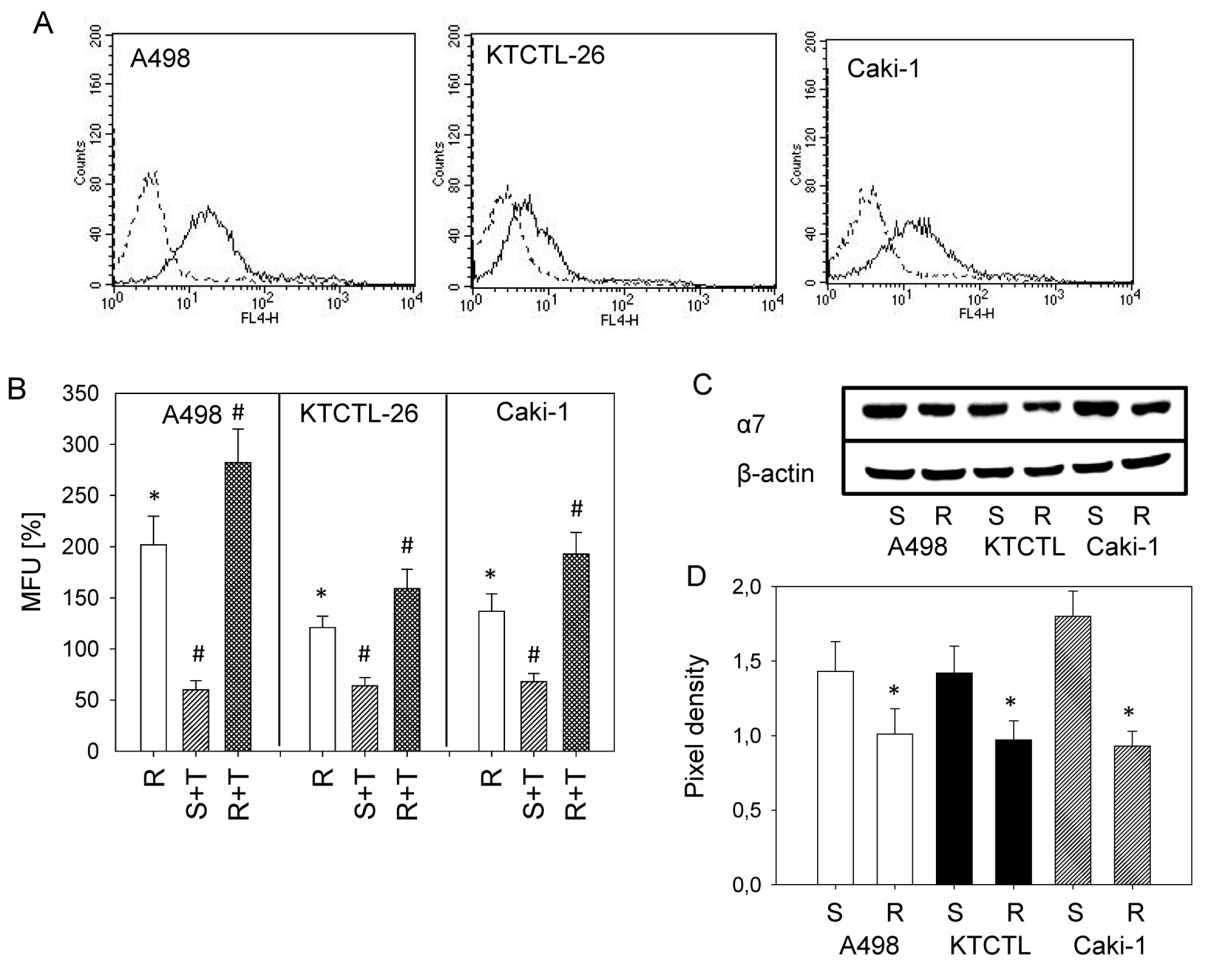
ITGA7 expression **(A)** FACS analysis of ITGA7 surface expression on sensitive A498, KTCTL-26, and Caki-1 cells. Dotted lines show background staining using a goat anti-mouse IgG1-PE. One of three independent experiments. **(B)** Evaluation of ITGA7 specific mean fluorescence (MFU) in the resistant (R) cells, in resistant cells reexposed to 10 nM temsirolimus (“R+T”), and sensitive cells treated with 10 nM temsirolimus (S+T). MFU values measured after 24 h temsirolimus exposure were compared to untreated controls set to 100%. (100% controls are not shown in the figure). ^*^indicates significant difference to these sensitive controls. #indicates significant difference between temsirolimus treated and non-treated cells. **(C)** Modification of the intracellular ITGA7 protein level. β-actin served as internal control. One representative of three separate experiments is shown. **(D)** Pixel density analysis of the protein bands to calculate the ratio of protein intensity/β-actin intensity. ^*^indicates significant difference between resistant and sensitive cells.

ITGA7 was expressed on the cell surface of the drug-sensitive Caki-1, KTCTL-26, and A498 cells as demonstrated by flow cytometry (Figure 4A). ITGA7 surface expression level was elevated in the temsirolimus-resistant cells, compared to sensitive cells set to 100% and further increased following reexposure of the resistant cells to 10 nM/ml temsirolimus (Figure 4B). In contrast, treating sensitive tumor cells with temsirolimus induced a significant loss of cell surface ITGA7. Intracellular ITGA7 was significantly lower in all three temsirolimus-resistant cell lines, compared to sensitive cells (Figure 4C, 4D).

### ITGA7 is involved in adhesion and chemotaxis regulation

**Figure 5:**
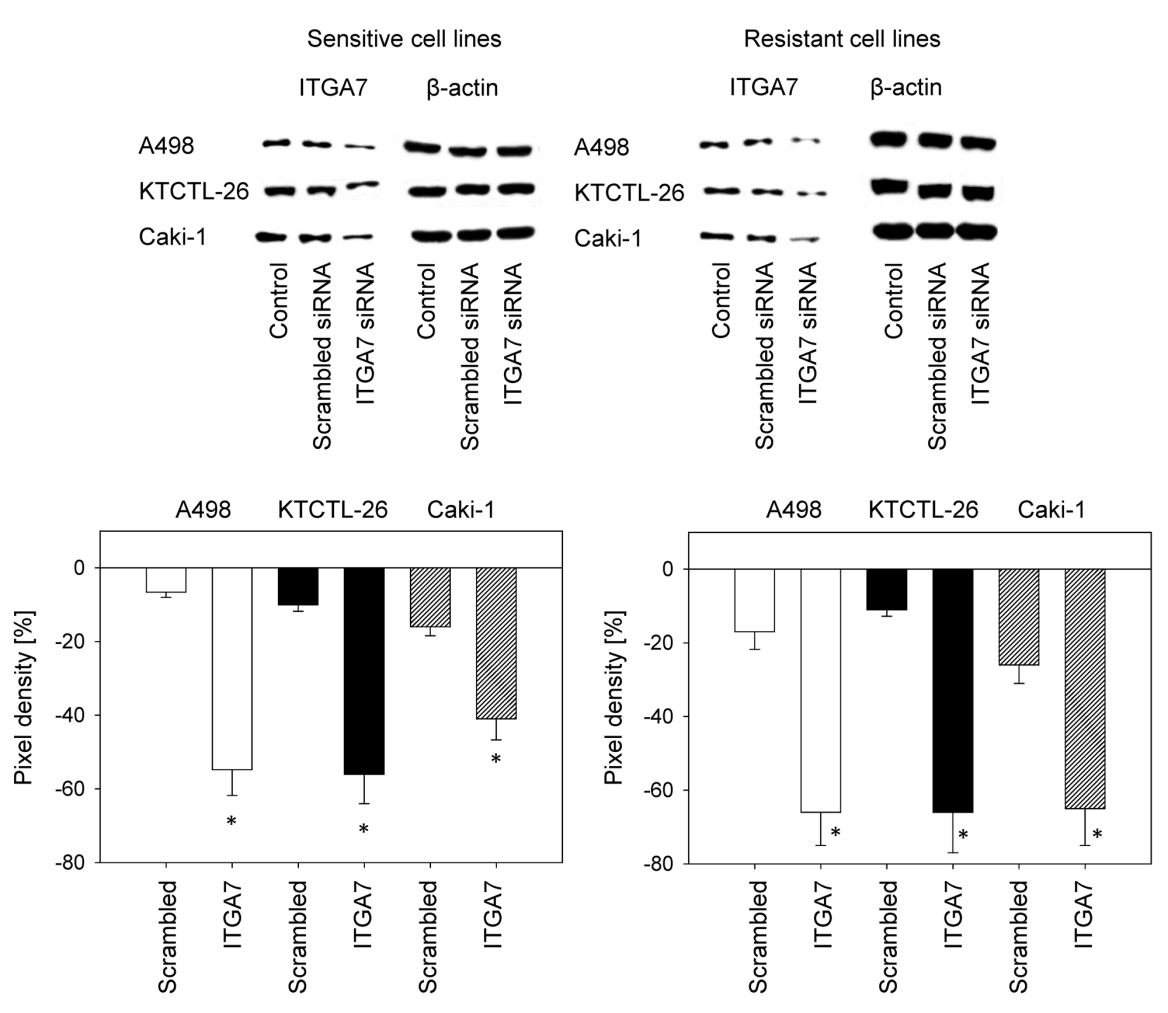
Confirmation of siRNA blockade of ITGA7 Resistant and sensitive A498, KTCTL-26, and Caki-1 cells were transfected with ITGA7 siRNA. One representative of three experiments is shown. Pixel density analysis of the protein bands was done to calculate percentage difference between controls and crambled siRNA and between controls and ITGA7 siRNA. Controls were set to 100%. ^*^indicates significant difference.

To explore the functional relevance of ITGA7, the protein was knocked down in Caki-1, KTCTL-26, and A498 cells and adhesion and chemotaxis experiments were carried out. Control studies demonstrated that specific ITGA7 down-regulation in the temsirolimus-sensitive as well as the temsirolimus-resistant tumor cells occurred (Figure 5).

**Figure 6:**
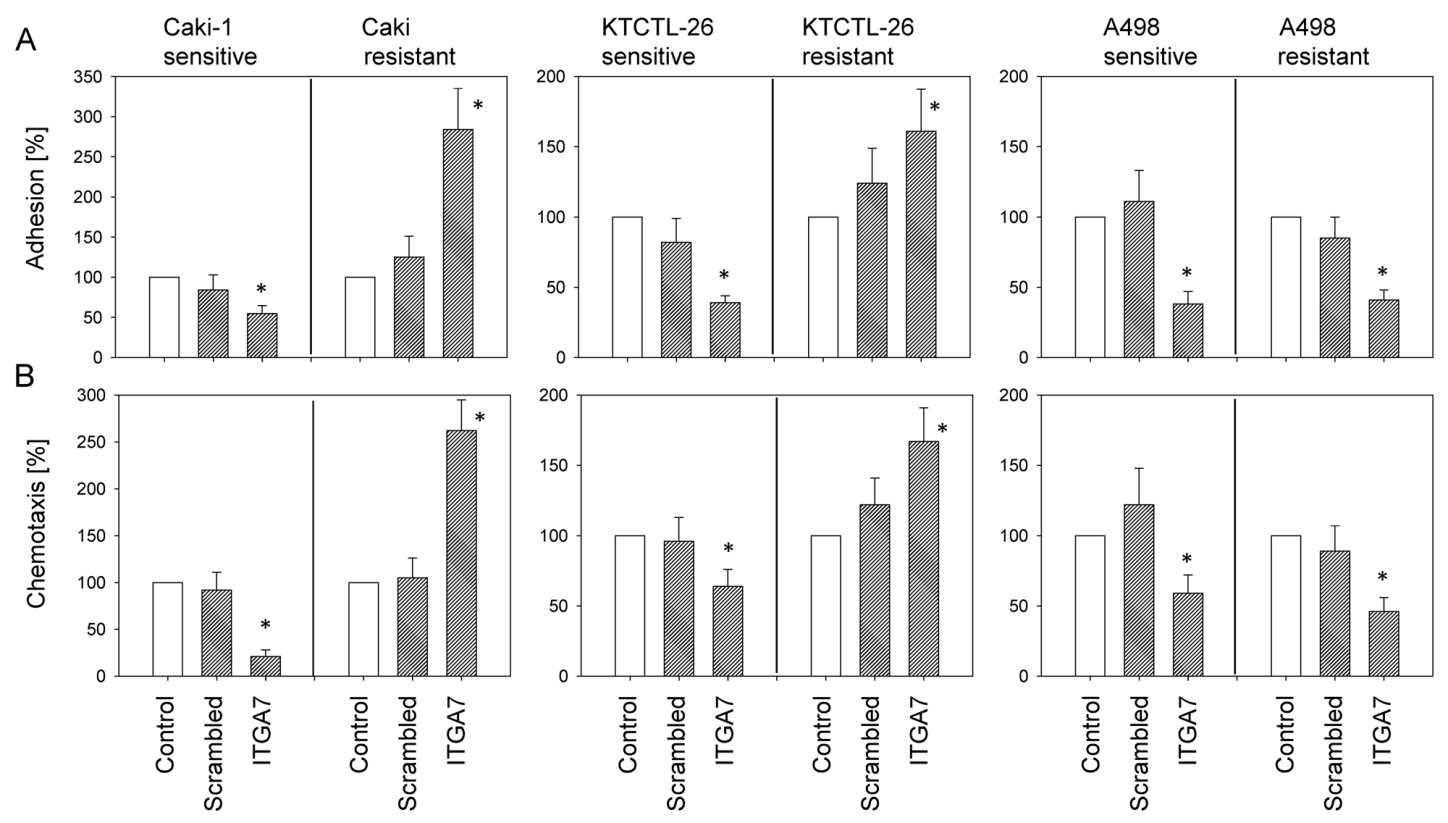
Influence of ITGA7 knock-down on tumor cell adhesion **(A)** and chemotaxis **(B)**. Untreated cells and with control siRNA transfected cells served as controls. Cell number of control adhesion and chemotaxis was set to 100%. ^*^Indicates significant difference to untreated controls (n = 6).

Loss of ITGA7 diminished adhesion of the temsirolimus-sensitive but elevated adhesion of the temsirolimus-resistant Caki-1 and KTCTL-26 cells, each compared to respective controls. In the A498 model, adhesion of both temsirolimus-sensitive and temsirolimus-resistant cells was diminished following ITGA7 knock-down (Figure 6A). Likewise, ITGA7 loss suppressed chemotaxis of temsirolimus-sensitive Caki-1 and KTCTL-26 cells, but enhanced chemotaxis in temsirolimus-resistant Caki-1 and KTCTL-26 cells. In the A498 model, chemotaxis of both temsirolimus-sensitive and temsirolimus-resistant cells was diminished following ITGA7 knock-down (Figure 6B).

**Figure 7:**
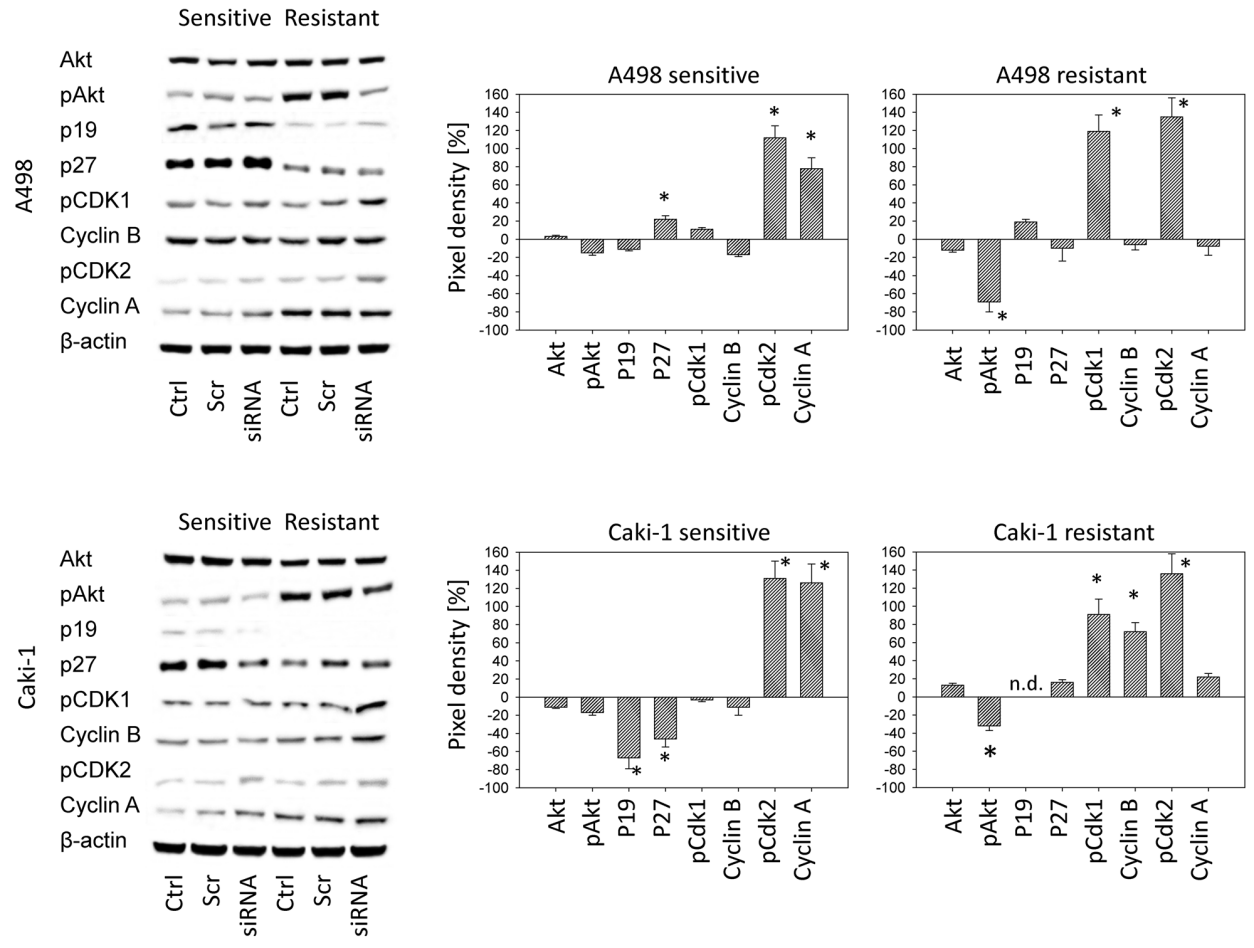
Left panel: Western blot analysis of the protein expression profile in drug-sensitive versus drug-resistant tumor cells following siRNA knock down of ITGA7 Ctrl: control values, Scr: scrambled siRNA, siRNA: ITGA7 siRNA. Right panel: Pixel density analysis of the protein bands. The figure shows percentage difference between controls and ITGA7 siRNA. Controls were set to 100%. ^*^indicates significant difference to the controls.

ITGA7 dependent cell signaling was also analyzed. In the drug sensitive A498 cell line, ITGA7 knock down caused a distinct p27 elevation and moderately enhanced pCdk2 and Cyclin A. In the resistant A498 subline, pCdk1 and pCdk2 were strongly elevated, whereas pAkt was considerably diminished following ITGA7 knock down (Figure 7). Similarly to A498 cells, Caki-1 cells showed enhanced pCdk1 (drug resistant cells) and pCdk2 (both drug-sensitive and drug-resistant cells) expression. Cyclin A was elevated in the sensitive, and pAkt was lowered in the resistant Caki-1 cells. Differences to A498 cells were seen in that p19 and p27 were reduced in sensitive, and Cyclin B was up-regulated in resistant Caki-1 (Figure 7).

### Clinical correlation

**Figure 8:**
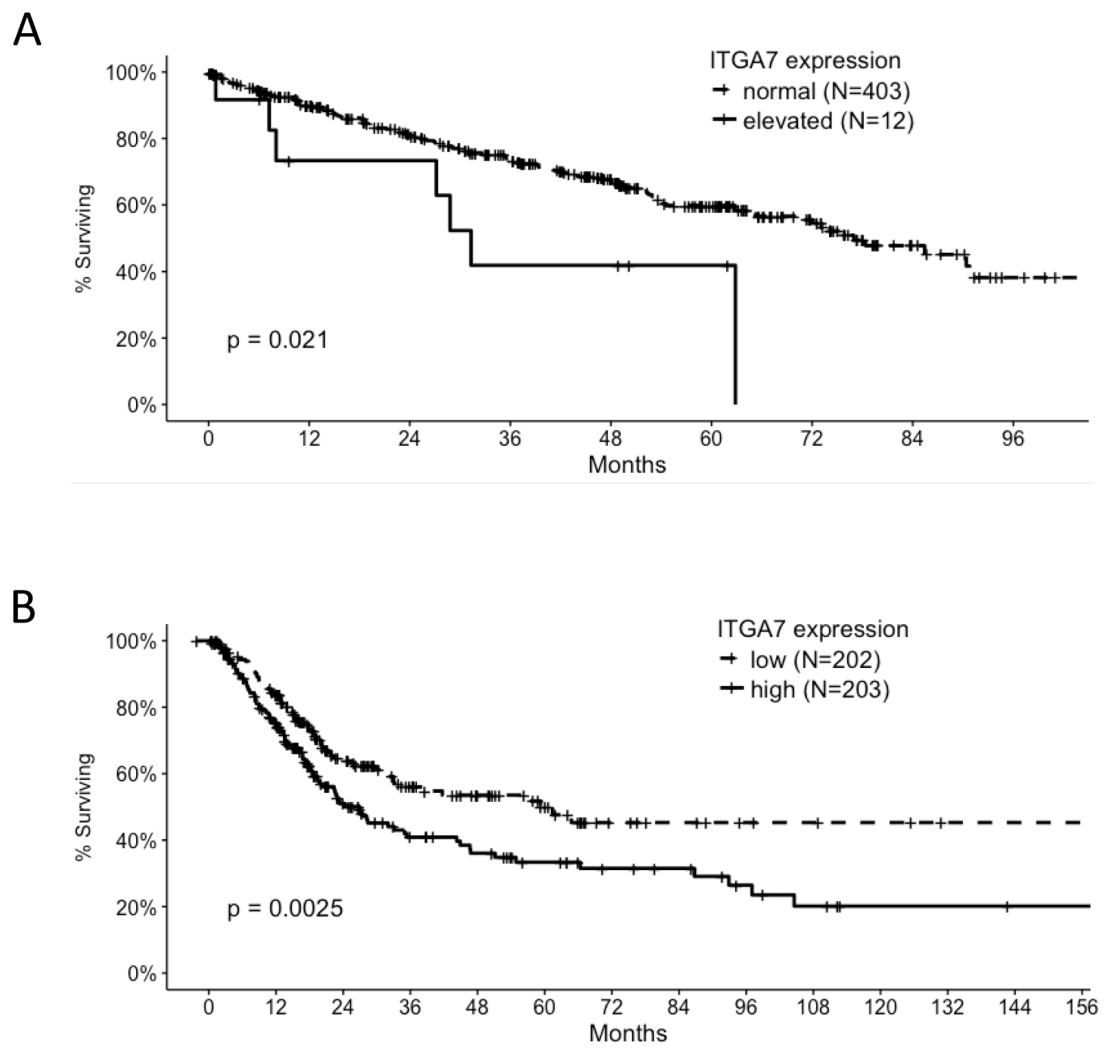
Correlation of ITGA7 expression and overall survival of clear cell renal carcinoma **(A)** and bladder cancer patients **(B)** Data were derived from the TCGA database.

Our findings are supported by TCGA data and other datasets available in Oncomine. ITGA7 was found to be significantly over-expressed in paired samples from clear cell renal cell carcinoma compared to adjacent normal tissue in multiple independent studies ([10-14] and was elevated in all renal cell carcinoma studies available in Oncomine. High ITGA7 expression (z score >= 2) was significantly associated with poor overall survival of clear cell renal carcinoma patients [13]. Elevated ITGA7 expression levels were also significantly associated with poor overall survival of bladder urothelial carcinoma patients (Figure 8) [15].

## DISCUSSION

Temsirolimus has significantly improved progression free survival and, for a minority of RCC patients, overall survival. However, the benefit of temsirolimus treatment is temporary, and rapid disease progression under chronic treatment develops. The mechanism behind acquired resistance is not fully understood. Cell culture studies point to feedback activation of the PI3K-Akt signaling pathway, caused by inhibiting the mTOR complex 1 (mTORC1) [16]. Morphoproteomic analysis of biopsies from RCC patients who clinically progress despite temsirolimus or everolimus therapy has revealed constitutively activated STAT3 and ERK pathways in collaboration with the mTOR complex 2 (mTORC2) and Akt [17].

Recently, it has been shown that integrin α5 (ITGA5) and β3 (ITGB3) adhesion receptors become modified in RCC cells during resistance development, leading to activation of the cellular motor machinery [18]. Such activation may explain why RCC not only triggers re-growth under mTOR-inhibitor therapy but also accelerates metastatic dissemination. The present data point to a close association between ITGA7 expression and temsirolimus-resistance with pronounced up-regulation of ITGA7 surface expression in temsirolimus-resistant RCC (particularly following reexposure to temsirolimus). Cytoplasmic ITGA7 content was diminished in resistant compared to sensitive tumor cells. Therefore, we postulate that temsirolimus-resistance is paralleled by ITGA7 translocation from inside the cell to the outer cell surface. The phenomenon is not unique since integrin trafficking from and to the plasma membrane has been demonstrated to be a crucial mechanism in regulating cell motility and invasion [19].

Jacquemet et al. have postulated that the kind of cell activation (at least partially) depends on integrin localization [20]. In good accordance, it has been shown that transmembranous shifting of the integrin subtype β1 (ITGB1) may contribute to metastatic progression of bladder cancer cells [21], and ITGB3 translocation has been associated with adhesive behaviour in prostate cancer cells [22].

Western blotting documented a reduced ITGA7 protein level in temsirolimus-resistant cancer cells. To explore functional relevance, ITGA7 was knocked down and adhesion and migration experiments were carried out. Loss of ITGA7 protein was associated with reduced adhesion and chemotaxis of temsirolimus-sensitive Caki-1 and KTCTL-26 cells, but with enhanced adhesion and motility in temsirolimus-resistant cell lines. Previous studies [5, 18] support the assumption that alteration of the ITGA7 expression level and integrin redistribution processes, observed during long-term treatment, might be coupled to functional changes of the integrin molecule, driving resistant tumor cells to high motility. Both Caki-1 and KTCTL-26 displayed higher chemotactic activity when acquired resistance had developed. Furthermore, treating sensitive tumor cells with temsirolimus diminished chemotaxis, whereas re-treatment of the resistant cells with low-dosed temsirolimus enhanced chemotaxis.

The underlying molecular mode of action can only be speculated upon. Flevaris et al. and Deshmukh et al. have provided a working hypothesis where the integrin function may depend on the structure and folding of the cytoplasmic integrin tail, shifting the integrin influence to different signaling pathways [23, 24]. Whether the integrin shift observed in the present model is accompanied by structural protein modification has not been addressed and requires further investigation. Since the ITGA7 protein was knocked down but did not specifically block the membranous ITGA7 receptor (a function blocking ITGA7 monoclonal antibody was not available at the time), it is not possible to determine whether the diminished intracellular ITGA7 content altered inside-out-signaling. Elevated membranous ITGA7 could have altered outside-in-signaling, or both processes could contribute to the altered signal. The negative correlation between cytoplasmic ITGA7 protein level and RCC adhesion and migration properties of Caki-1 and KTCTL-26 indicate that ITGA7 is a tumor suppressor. However, if ITGA7 surface expression dominates, the opposite may hold true and ITGA7 may serve as an oncoprotein.

Whatever the exact mechanism, the results presented here indicate considerable ITGA7 traffic from inside the RCC cell to the outer membrane, which not only elevates tumor cell–matrix-interaction but also causes temsirolimus-resistant cells to become highly motile upon reexposure to temsirolimus. This could occur either by down-regulation of tumor suppressive signals or by activating oncogenic pathways.

ITGA7 function was comparable in both sensitive and resistant A498 cells, evidenced by siRNA knock-down studies. A conversion of ITGA7’s function, observed in resistant Caki-1 and KTCTL-26 cells under temsirolimus reexposure, did not take place in A498 cells. Another mode of action must therefore occur in A498 cells, accounting for this phenomenon. The integrin subtypes ITGA5 and ITGB3, as critical modulators of RCC metastasis once resistance towards temsirolimus occurs [18], has been established. Therefore, regulation of tumor cell invasiveness cannot exclusively be ascribed to ITGA7, but involves other integrins. A negative correlation between ITGA7 and ITGA5 has recently been reported, showing that a high ITGA7 surface expression induces blockage of ITGA5 function [25]. Whether this negative feedback mechanism counteracts ITGA7 driven elevation of chemotaxis in the temsirolimus-resistant A498 cells is, however, unclear.

Since Caki-1 and KTCTL-26 are derived from clear-cell RCC, whereas A498 is derived from non-clear RCC, the role of ITGA7 in adhesion and migration might depend on the RCC subtype. Based on a mouse model of Duchenne muscular dystrophy, ITGA7 was shown to cross-communicate with the Akt-mTOR-axis, leading to signal amplification and growth promotion [26]. This investigation points to a further aspect of ITGA7’s function. Whether ITGA7 activates the mitotic cycle in A498 cells during resistance development, influencing A498 chemotaxis, remains unclear and requires further investigation.

pAkt was suppressed in the drug-resistant tumor cell lines, particularly in A498 cells, following ITGA7 knock down, whereas pCdk1 and pCdk2 were elevated. Zhang and coworkers have reported that Akt signaling correlates with the metastatic behaviour of tumor cells [27]. In the present investigation a negative association was seen between ITGA7 expression and activity of the cell cycle regulating proteins Cdk1 and Cdk2. Therefore, it seems likely that ITGA7 switches Akt-dependent pathways during resistance development so that the cellular motor machinery becomes activated to suppress cell proliferation. This assumption is speculative. However, Riggio et al. recently reported that Akt controls tumor cell growth and invasion in an opposing manner, i.e. reduced proliferation is coupled to enhanced metastatic potential and vice versa [28]. Furthermore, *in vitro* experiments on RCC cell lines have shown that integrins undergo functional change during chronic temsirolimus treatment, forcing RCC invasion [18].

The western blot data presented here, may help explain how this functional switch takes place. ITGA7 translocation to the outer cell membrane could cause altered intracellular signaling, presumably via Akt, leading to de-activation of growth promoting pathways (pCdk1, pCdk2) and activating invasion promoting pathways. The Akt antibody employed in this investigation did not discriminate between Akt isoforms. It is therefore not possible to exclude the possibility that Akt 1 and Akt 2 separately activate either cell proliferation (via Akt 1) or cell metastasis (via Akt 2), as has been speculated by others [28, 29]. Preincubation with an anti-beta1 integrin function blocking antibody suppressed Akt 2 caused cell invasion *in vitro* [30]. Further investigation is now required to establish whether ITGA7 induces a switch from Akt 1 to Akt 2 signaling during resistance development.

Independent on how Akt may be influenced by ITGA7, evidence is provided here that ITGA7 is involved in adhesion and migration of the three RCC cell lines, Caki-1, KTCTL-26, and A498. ITGA7 translocation, observed under temsirolimus-resistance, contributes to significant acceleration of chemotactic movement of the two clear cell RCC lines, Caki-1, KTCTL-26.

The role of ITGA7 is controversial. High ITGA7 surface expression has been negatively correlated with glioblastoma patient survival, and targeting ITGA7 by RNAi has been shown to lead to a significant reduction of tumor cell invasion *in vitro* [6]. These findings accord well with the present investigation where an increased amount of ITGA7 on the cell surface is associated with increased chemotaxis in temsirolimus-sensitive cells. Still, an opposite function of ITGA7 has been described by other investigators. Immunohistochemical analysis of human malignant pleural mesothelioma tissue sections have shown a positive correlation between ITGA7 expression and median overall survival, and migratory activity of tumor cells with a high ITGA7 mRNA level was diminished, compared to cells with a low ITGA7 mRNA level [7]. However, these investigators did not evaluate cytoplasmic ITGA7 proteins and ITGA7 surface expression, making final assessment difficult. Hypothetically, a high mRNA level might be associated with a high ITGA7 level, but with low membrane expression.

Tan et al. has suggested that ITGA7 plays a role as a tumor-suppressor with a high intracellular content and is associated with growth inhibition of prostate cancer cells [31]. This is similar to the observation that ITGA7 protein content negatively correlates with RCC adhesion and migration. The tissue inhibitor of metalloproteinases-3 (TIMP3) has been identified as a relevant downstream target of ITGA7 [31]. This is important, since TIMP3 is a potent inhibitor of matrix metalloproteinases (MMP), relevant for preventing invasion and migration of RCC cells [32]. Therefore, the reduced ITGA7 protein level, detected in temsirolimus-resistant Caki-1 and KTCTL-26 cells, may be interpreted in such way that ITGA7-TIMP3-interaction is down-regulated during resistance development, linking this interaction to invasive activation. In addition, up-regulation of ITGA7 along the cell surface may enhance activation of oncogenic pathways. Evaluation of MMPs and TIMPs in RCCs have revealed high levels, particularly in clear-cell RCCs [33], which might explain why a functional switch of ITGA7 action on adhesion and migration was observed in Caki-1 and KTCTL-26 (clear-cell subtype) but not in A498 (non-clear cell subtype). Since MMP and TIMP profiles in these cell lines were not compared, this explanation remains speculative. Further investigation into this aspect is required.

The study on glioblastoma cells carried out by Haas et al. [6] identified ITGA7 as a glioblastoma biomarker and candidate therapeutic target. Although ITGA7 has been demonstrated to be involved in metastatic behaviour promoted by temsirolimus-resistance in the present investigation, ITGA7 expression must be carefully monitored in patients before finally assessing its role as a target in treating RCC.

It must be emphasized that the data provided here is based on *in vitro* model. However, a recent publication, based on tumor tissue derived from hepatocellular carcinoma patients and from an animal model, reports that activated Akt signaling, a typical feature of temsirolimus resistance [2], may correlate with ITGA7 expression and tumor metastasis [27]. Ongoing experiments are now directed at concentrating on the function of ITGA7 in a mouse xenograft model to verify our *in vitro* results.

## MATERIALS AND METHODS

### Cell culture

Caki-1 and KTCTL-26 cells were purchased from LGC Promochem (Wesel, Germany) and A498 cells from Cell Lines Service (Heidelberg, Germany). Tumor cells were grown and subcultured in RPMI 1640 medium (Seromed, Berlin, Germany) supplemented with 10% fetal calf serum (FCS), 20 mM HEPES-buffer, 1% glutamax, and 1% penicillin/streptomycin (all: Gibco/Invitrogen; Karlsruhe, Germany) at 37°C in a humidified, 5% CO_2_ incubator. Subcultures from passages 5–24 were employed.

Human umbilical vein endothelial cells (HUVEC) were harvested by enzymatic treatment with dispase (Gibco/Invitrogen) and cultured in Medium 199 (M199; Biozol, Munich, Germany), supplemented with 10% FCS, 10% pooled human serum, 20 μg/ml endothelial cell growth factor (Boehringer, Mannheim, Germany), 0.1% heparin, 100 ng/ml gentamycin, and 20 mM HEPES-buffer (pH 7.4). Subcultures from passages 2–6 were employed.

### Resistance induction, drug treatment

Temsirolimus (LC Laboratories, Woburn, MA, USA) was dissolved in DMSO as a 10 mM stock solution and stored as aliquots at −20°C. Before experiments, temsirolimus was diluted in cell culture medium to the final concentration. The temsirolimus-resistant RCC sublines were established over 12 months by exposing parental cells to temsirolimus, starting at 1 nM/ml and increasing stepwise to 1 μM/ml. Cell culture medium alone was applied to control cell cultures. Resistance was defined by an increased IC_50_ value of the temsirolimus-resistant versus temsirolimus-sensitive cells (A498: 5fold; KTCTL-26: 60fold; Caki-1: 22fold [18]).

To analyze the influence of temsirolimus on adhesion and chemotactic movement of the resistant versus the sensitive tumor cell lines (see below), cell culture medium of the temsirolimus-resistant Caki-1, KTCTL-26, or A498 cells containing 1 μM temsirolimus was replaced by temsirolimus-free medium to avoid unspecific effects. A medium change was also carried out in the temsirolimus-sensitive cell culture system. After 3 days, 10 nM/ml temsirolimus was added to both resistant and sensitive cells (controls received fresh medium without temsirolimus), and adhesion and chemotactic movement were analyzed. To exclude toxic effects of temsirolimus, cell viability was determined by trypan blue (Gibco/Invitrogen).

### Adhesion to HUVEC and to immobilized matrix proteins

To analyze tumor cell–endothelial cell-interaction, HUVEC were transferred to six-well multiplates (Falcon Primaria; BD Biosciences, Heidelberg, Germany). When confluency was reached, RCC cells (temsirolimus-resistant and temsirolimus-sensitive) were detached from their culture flasks by Accutase treatment (PAA Laboratories, Cölbe, Germany). Cells (0.5 × 10^6^) were then added to the HUVEC monolayer for 30, 60, or 120 minutes. Subsequently, nonadherent tumor cells were washed off using warmed (37°C) M199. The remaining cells were fixed with 1% glutaraldehyde. Adherent tumor cells were counted in five different fields of a defined size (5 × 0.25 mm^2^) using a phase-contrast microscope, and the mean cellular adhesion rate was calculated.

To evaluate tumor cell binding to immobilized matrix proteins, six-well multiplates (Falcon Primaria) were coated with collagen G [consisting of 90% collagen type I and 10% collagen type III; diluted to 400 μg/ml in phosphate-buffered saline (PBS); Seromed, Berlin, Germany] or fibronectin (diluted to 50 μg/ml in PBS; BD Biosciences) overnight. Unspecific cell binding was evaluated using culture plates treated with Poly-D-Lysine (Nunc, Wiesbaden, Germany). Plastic dishes served as the background control. Plates were washed with 1% BSA in PBS to block nonspecific cell adhesion. Tumor cells (0.5 x 10^6^) were then added to each well for 60 minutes. Subsequently, nonadherent tumor cells were washed off, and the remaining adherent cells were fixed with 1% glutaraldehyde and counted under a microscope. The mean cellular adhesion rate, defined by adherent cells_coated well_ − adherent cells_background_, was calculated from five different observation fields (5 × 0.25 mm^2^).

### Chemotaxis

Serum-induced chemotactic movement was investigated using six-well Transwell chambers (Greiner Bio-One, Frickenhausen, Germany) with 8-μm pores. 0.5 × 10^6^ Caki-1, KTCTL-26, or A498 cells per milliliter (resistant versus sensitive) were placed in the upper chamber in serum-free medium. The lower chamber contained 10% serum. After a 20 h incubation, the upper surface of the Transwell membrane was gently wiped with a cotton swab to remove nonmigrating cells. Cells, which had moved to the lower surface of the membrane, were stained using hematoxylin and counted under a microscope. The mean chemotaxis rate was calculated from five different observation fields (5 × 0.25 mm^2^).

### ITGA7 surface expression

RCC cell lines were detached from their culture flasks by Accutase (PAA Laboratories GmbH, Pasching, Austria) and washed in blocking solution (PBS, 0.5% BSA). The cells were then incubated for 60 minutes at 4°C with a phycoerythrin (PE)-conjugated monoclonal antibody directed against ITGA7 (IgG1, clone 3C12; Abcam, Cambridge, UK). Integrin expression was then measured using a FACScan (BD Biosciences; FL-2H (log) channel histogram analysis; 1 × 10^4^ cells per scan) and expressed as mean fluorescence units. A mouse IgG1-PE (MOPC-21; BD Biosciences) was used as an isotype control.

### Western blot analysis

To investigate the ITGA7 protein level in Caki-1, KTCTL-26, and A498 cells (resistant versus sensitive), tumor cell lysates were applied to a 7% polyacrylamide gel and electrophoresed for 90 minutes at 100 V. The protein was then transferred to nitrocellulose membranes (1 h, 100 V). After blocking with nonfat dry milk for 1 h, the membranes were incubated overnight with the ITGA7 monoclonal antibody (IgG2a, clone 8G2; Bio-Rad, München, Germany). To evaluate ITGA7 dependent signaling, the following antibodies were used: anti-cyclin A (IgG1, clone 25), anti-cyclin B (IgG1, clone 18), anti-Akt (IgG1, clone 55), anti-phospho Akt (pAkt; clone 104A282, IgG1), anti-p27 (IgG1, clone 57; all: BD Biosciences, Heidelberg, Germany), anti-p19 INK4d (IgG, polyclonal; ThermoFisher Scientific, Dreieich, Germany), anti-phospho-Cdk1 (pY15; IgG1, clone 44), anti-phospho-Cdk2 (Thr160; both: MerckMillipore, Darmstadt, Germany). HRP-conjugated goat anti-mouse IgG (1:5.000; Upstate Biotechnology, Lake Placid, NY) served as the secondary antibody. The membranes were briefly incubated with ECL detection reagent (ECL; Amersham/GE Healthcare, München, Germany) to visualize the proteins and then analyzed by the Fusion FX7 system (Peqlab, Erlangen, Germany). β-Actin (1:1.000; clone AC-15; Sigma-Aldrich, Taufenkirchen, Germany) served as the internal control. Gimp 2.8 software was used to perform pixel density analysis of the protein bands and to calculate the ratio of protein intensity/β-actin intensity.

### Knock-down studies

RCC cell lines (3 × 10^5^ per well) were transfected with small interfering RNA (siRNA) directed against ITGA7 (1 nmol, HS_ITGA7_10 FlexiTube siRNA: SI05137398; Qiagen, Hilden, Germany) with a siRNA/transfection reagent (HiPerFect Transfection Reagent; Qiagen) ratio of 1:6. Untreated cells and cells treated with 1 nM control siRNA (AllStars Negative Control siRNA; Qiagen) served as controls. Subsequently, tumor cell chemotaxis was analyzed as indicated above.

### Clinical data

RNA sequencing data (normalized RSEM values and z values) from the TCGA-KIRC [13] and TCGA-BLCA project [15] analyzed on the IlluminaHiSeq_RNASeqV2 platform and clinical data were accessed via the cBioPortal interface on 24.01.2018. Clinical data included in the analysis were vital status and time-to-event (equals to time to death if patient deceased, or to time to last follow up if alive). Cases with right-censored time-to-event data were included in the analysis. Data was analyzed using R and graphs were created with ggplot2. The log-rank test implemented in R’s survdiff package was used to test for a between-group difference in survival probability and to calculate a p value.

### Statistics

All experiments were performed three to six times. Statistical significance was calculated with the Wilcoxon–Mann-Whitney U test. Differences were considered statistically significant at a P value less than 0.05.
